# Fasting mitigates immediate hypersensitivity: a pivotal role of endogenous D-beta-hydroxybutyrate

**DOI:** 10.1186/1743-7075-11-40

**Published:** 2014-08-28

**Authors:** Shigeru Nakamura, Ryuji Hisamura, Sachiko Shimoda, Izumi Shibuya, Kazuo Tsubota

**Affiliations:** 1 Department of Ophthalmology, Keio University School of Medicine, 35 Shinanomachi, Shinjyuku, Tokyo 160-8582, Japan; 2 Research center, Ophtecs Corporation, Hyogo, Japan; 3 Department of Veterinary Physiology, Faculty of Agriculture, Tottori University, Tottori, Japan; 4 Health Science Laboratory, Keio Research Institute at SFC, Keio University, Kanagawa, Japan

## Abstract

**Background:**

Fasting is a rigorous type of dietary restriction that is associate with a number of health benefits. During fasting, ketone bodies significantly increase in blood and become major body fuels, thereby sparing glucose. In the present study, we investigated effects of fasting on hypersensitivity. In addition, we also investigated the possible role of D-beta-hydroxybutyrate provoked by fasting in the attenuation of immediate hypersensitivity by fasting.

**Methods:**

Effects of fasting on systemic anaphylaxis were examined using rat model of toluene 2, 4-diisocyanate induced nasal allergy. In addition to food restriction, a ketogenic high-fat and low-carbohydrate diet that accelerates fatty acid oxidation and systemic instillation of D-beta-hydroxybutyrate were employed to elevate internal D-beta-hydroxybutyrate concentration. We assessed relationship between degranulation of rat peritoneal mast cells and internal D-beta-hydroxybutyrate concentration in each treatment. Changes in [Ca^2+^]i responses to compound 48/80 were analyzed in fura 2-loaded rat peritoneal mast cells derived from the ketogenic diet and fasting.

**Results:**

Immediate hypersensitivity reaction was significantly suppressed by fasting. A significant reduction in mast cells degranulation, induced by mast cell activator compound 48/80, was observed in rat peritoneal mast cells delivered from the 24 hours fasting treatment. In addition, mast cells delivered from a ketogenic diet and D-beta-hydroxybutyrate infusion treatment also had reduced mast cell degranulation and systemic D-beta-hydroxybutyrate concentrations were elevated to similar extent as the fasting state. The peak increase in [Ca^2+^]i was significantly lower in the ketogenic diet and fasting group than that in the control diet group.

**Conclusions:**

The results of the present study demonstrates that fasting suppress hypersensitivity reaction, and indicate that increased level of D-beta-hydroxybutyrate by fasting plays an important role, via the stabilization of mast cells, in suppression of hypersensitivity reaction.

## Background

Dietary restriction, now usually referred to as caloric restriction, has been known about for decades and is a very robust intervention that produces a number of associated health benefits and ameliorates diseases with aging [1]. Fasting is defined as voluntary food deprivation that has been performed as religious observance for spiritual development and promotion of health among different people and religions worldwide [2]. Fasting have been used mainly for the treatment of obesity [3,4], and its therapeutic effects have also been reported to ameliorate hypertension [5], epilepsy [6], psychosomatic disease [7], and bronchial asthma [8]. However, the mechanisms for these benefits are still largely unknown.

During fasting, the body begins to break itself down to supply energy and dynamic metabolic changes occur [9,10]. At the early stage of fasting, 1-2 days after food deprivation, hepatic glycogen and amino acids derived from muscle began to serve as the main source for glycogenesis for energy supply. After sources for hepatic glycogenesis storage have been depleted, 3 days to 1 week later, glycerol and fatty acids released from adipose tissues become the major source of energy production. Generated fatty acids are converted to acetyl-coA and are shunted to the ketogenic pathway resulting in the production of ketone bodies mainly in the liver. These ketones are transported to peripheral tissues where they can be converted back to acetyl CoA and utilized in the tricarboxylic acid [TCA] cycle for energy supplementation. In this condition, serum ketone body concentrations increases approximately 1000 fold from the normal state [11].

The beneficial pleiotropic effects of ketone bodies have been reported other than the role of metabolic fuel. D-beta-hydroxybutyrate (D-BHB), a major circulating ketone body, has been demonstrated to be effective in neurological disorders such as Alzheimer’s, Parkinson’s, and Huntington’s disease in animal models and humans [12-14]. In addition, D-BHB has been found to attenuate corneal disorder [15,16], retinal degeneration [17], and bone loss from ovariectomy [18]. Recent finding also identified that D-BHB is an endogenous histone deacetylases inhibitor and helps cells resist oxidative stress [19]. These evidences raised the hypothesis that increased concentrations of ketone bodies may have a possible role in the health benefits obtained during food deprivation.

Several studies have shown that fasting enhances immunological defenses. Short-term fasting resulted in lower levels of antigen-specific IgE and attenuated pulmonary inflammation in a rat model of allergic responses to the house dust mite [20]. The Guinea pigs sensitized with the ovalbumin reduced antigen-induced bronchoconstriction after fasting [21].

In the present study, we investigated effects of fasting on hypersensitivity using rat model of TDI induced nasal allergy. In addition, we also explored the possible role of D-BHB on the attenuation of immediate hypersensitivity by fasting. We showed that increases in serum D-BHB concentrations by fasting were strongly associated with suppression of immediate hypersensitivity and mast cell degranulation, which is known to initiate a hypersensitivity reaction [22].

## Materials and methods

### Animals

All animal experiments were performed under the following conditions; Room temperature 23 ± 2°C, relative humidity 60% ± 10%, and an alternating 12-hours light–dark cycle (8 AM to 8 PM). Animals were quarantined and acclimatized before experiments at least for a 1-week period standard diet (Oriental Yeast Industries, Tokyo, Japan) and water were available ad libitum. The care and handling of the animals were in accordance with the National Institute of Health guidelines. All procedures involving animals were carried out in accordance with the Institutional Guidelines.

### Fasting

For fasting treatment, mice and rats had access to only water. For the ketogenic diet, Diet D10070801 (Research Diets, New Brunswick, NJ, USA), consisting of 67% fat, 17% protein, and 0% carbohydrate was used.

### Rat model of toluene 2, 4-diisocyanate (TDI) induced Nasal Allergy

Nasal Allergy models were performed by the method described previously with slight modifications [23]. Six-week-old male Brown Norway rats (n = 6–8 in each experiment) were purchased from Japan SLC, Inc. Five μl of a 10% solution of TDI (Sigma-Aldrich, St. Louis, MO, USA) in ethyl acetate (Sigma-Aldrich, St. Louis, MO, USA) was intranasally applied to the bilateral nasal cavities once a day for five consecutive days (sensitization). This sensitization procedure was then repeated after a 1-day interval. Seven days after the latest sensitization, and prior to being evaluated, 5 μl of 10% TDI solution was applied again to the nasal cavaties to provoke nasal allergy-like behaviors. Sensitized rats without provocation by TDI solution served as the control group.

Nasal allergy-like behaviors were evaluated by counting the amount of nasal rubbing and sneezing during a 20 minute period immediately after TDI application. Changes in nasal secretions were evaluated by measuring the weight of cotton thread 60 minutes after TDI application.

The total plasma IgE levels were determined after TDI application. Blood was collected from the abdominal aorta. Plasma samples were obtained by centrifugation and stored at -30°C until use. The total plasma IgE level was determined by using Rat IgE ELISA Kit (Shibayagi Co., Ltd, Gumma, Japan) according to the manufacturers instruction.

After nasal secretion was measured, rats were applied to histological examination of the nasal cavity and mucosa of maxilloturbinate. Rats were euthanized by cervical dislocation under anesthetized with sodium pentobarbital. The head was separated from the neck and the whole anterior face sections were prepared using a multipurpose cryosection preparation kit (Section Lab Co., Ltd, Hiroshima, Japan) according to the method of Kawamoto [24]. In brief, anterior face of tissue was frozen in isopentane (-160°C) cooled with dry ice and then freeze-embedded with SCEM compound (Section Lab Co., Ltd, Hiroshima, Japan). Twenty-μm thick sections were cut with cryomicrotome (CM3050S, Leica Co., Ltd, Heidelberg, Germany) and collected with cryofilm (Section Lab Co., Ltd, Hiroshima, Japan). For hematoxylin and eosin (H&E) and Toluidine blue (pH4.1) staining, tissue sections were fixed by 4% paraformaldehyde after drying section and mounted with SCMM (Section Lab Co., Ltd, Japan). For immunohistochemistry, the sections were incubated with a polyclonal antibody to mast cell tryptase (Santa Cruz, CA, USA) at 4°C overnight, followed by labeling with Alexa 488-conjugated goat anti-rabbit IgG (Life Technologies, Inc. Grand Island, NY, USA) and Hoechst 33342 (Dojindo Co., Ltd, Kumamoto, Japan) at RT for 30 min. These sections were observed with microscope (BZ-9000, Keyence Co., Ltd., Tokyo, Japan) or a confocal microscope (LSM 710, Carl Zeiss, Inc. Heidelberg, Germany).

### Passive cutaneous anaphylaxis (PCA)

Six-week old male Wistar rats (Japan Clea, Inc. Tokyo, Japan) were used (n = 7–27 in each time point). The homologous antiserum used was prepared according to the method described. Briefly, rats were sensitized by intramuscular injection of 0.2 ml of 10 mg/mL egg albumin (Sigma-Aldrich, St. Louis, MO, USA) to both femurs and intraperitoneal administration of Bordetellapertussis inactive microorganism suspension (B. pertussis, Kitasato Institute Research Center for Biologicals, Saitama, Japan). On day 14, animals were bled and antiserum was collected. Separated antiserum was stored at -80°C.

Rat homologous antiserum (100 μL) was injected into the shaved back skin of rats. After 24 h, sensitized rats were injected with 0.5 mL of mixed solution of 0.5% Evan blue (Fluka, Buchs, Switzerland) and 1% egg albumin (1:1) through the tail vein. Thirty minutes later, rats were sacrificed and the dye content of the shaved dorsal skin was determined quantitatively. Pieces of the dorsal skin containing extravasated dye were removed and soaked overnight in stoppard glass tubes containing 1 ml of mixed solution of 0.6 N H_3_PO_4_/acetone (13:5). The absorbance of supernatant was measured at 620 nm using a spectrophotometer and the concentration of the dye in the shaved dorsal skin (μg/site) was determined. Fasting duration was determined as the time between food removal and antigen challenge.

### Systemic anaphylactic shock

Ten-week old male BALB/c mice (Japan Clea, Inc. Tokyo, Japan) were used (n = 6–12 in each experiment). For the evaluation for IgE dependent anaphylaxis, mice were sensitized with an intraperitoneal injection of 125 μg egg albumin dissolved in aluminum gel. Seven days later, mice were challenged by intraperitoneal injection of 125 μg egg albumin dissolved in aluminum gel [25]. For IgE independent anaphylaxis, anaphylactic shock was evoked by intraperitoneal injection of 6.5 mg/kg compound 48/80 (Sigma-Aldrich, St. Louis, MO, USA) in saline solution [26]. Immediately after the antigen or compound 48/80 challenge, rectal temperature was measured with a digital thermometer (Tateyama kagaku industry Co.,Ltd, Toyama, Japan).

### Preparation of rat peritoneal mast cells

Eleven-week old male Sprague Dawley rats (Japan Clea, Inc. Tokyo, Japan) were used (n = 4 in each treatment). Rat peritoneal mast cells (RPMC) were collected by rinsing the peritoneal cavities of normal rats with 20 ml of mast cell medium (MCM, containing 150 mM NaC1, 3.7 mM KC1, 3.0 mM Na_2_HPO_4_, 3.5 mM KH_2_PO_4_, 5.6 mM dextrose,0.1% gelatin, 0.1% (w/v) bovine serum albumin) containing 10 U/ml heparin under anesthesia with pentobarbital. Peritoneal cells were sedimented twice at 50 x G for 7 minutes at 4°C and re-suspended in MCM. RPMC were then washed twice with siraganian buffer [119 mM NaCl, 5 mM KCl, 0.4 mM MgCl_2_, 25 mM piperazine-N,N0-bis(2-ethanesulfonic acid)(PIPES), pH 7.2] and resuspended in siraganian buffer. RPMC (5 × 10^4^).

### Degranulation assay

The degree of degranulation was determined by measuring the release of β-hexosaminidase [27]. The enzymatic activity of β-hexosaminidase in supernatants and sonicated disrupted cell pellets was measured with p-nitrophenyl N-acetyl-β-d-glucosaminide (Sigma-Aldrich, St. Louis, MO, USA) in 0.1 M sodium citrate (pH 4.5) for 60 minutes at 37°C. The reaction was stopped by the addition of 1 M Na_2_CO_3_ glycine. The release of the product, 4-p-nitrophenol, was detected by absorbance at 405 nm. The extent of degranulation was calculated by dividing 4-p-nitrophenol absorbance in the supernatant by the sum of absorbance in the supernatant and disrupted cell pellet.

To induce RPMC degranulation, cells were incubated with 1 μg/mL compound 48/80 at 37°C for 10 minutes. For the evaluation of the effect of D-BHB on mast cell degranulation, cells were preincubated with various concentrations of D-BHB for 10 minutes, and then incubated with compound 48/80.

### Measurement of intracellular calcium

The [Ca^2+^]_i_ in RPMC was measured with the fluorescent Ca^2+^ indicator, Fura-2/AM (Invitrogen, Carlsbad, CA, USA). Cells were plated onto glass bottom culture dishes. The cell was loaded with the indicator by incubation with 2.5 μM of Fura-2/AM in in Standard Solution (140 mM NaCl, 5 mM KCl, 1 mM MgCl_2_, 10 mM HEPES, 10 mM dextrose) for 4 h at room temperature (22–24°C). After loading, cells were washed three times with Standard solution and incubated for further 30 minutes to ensure de-esterification. Fluorescence recordings were performed using two set-ups, employing either video-imaging or a single detector system. For video-imaging, a coverslip with dye-loaded cells was mounted in a chamber fixed on the stage of an inverted fluorescence microscope (IX71, Olympus Co., Tokyo, Japan) equipped with UV objective lens (UApo 20X3/340, Olympus Co., Tokyo, Japan). The emission signal passed through a band pass filter (500 ± 10 nm) and was detected by a 12 bit CCD camera (C-8214, Hamamatsu Photonics K.K., Hamamatsu, Japan). Fluorescence intensities at 510 nm with excitation at 340 and 380 nm were recorded at 5 s intervals. The [Ca^2+^]_i_ in an individual cell was calculated from the ratio of fluorescence images acquired at excitation at 340 nm to those at 380 nm. All experiments were performed at room temperature (22–24°C).

Increases in [Ca^2+^]_i_ were evoked by 1 μg/mL compound 48/80 for 240 sec. For the evaluation of the effect of D-BHB on [Ca^2+^]_i_, cells were stimulated with compound 48/80 under 100 mM D-BHB.

### Serum and intraperitoneal D-BHB

Eleven-week old male Sprague Dawley rats (Japan Clea, Inc. Tokyo, Japan) were used (n = 4 in each treatment). The concentration of D-BHB in collected serum and intraperitoneal fluid was measured using Ketone test A (Sanwa kagaku kenkyusho Co., Ltd., Nagoya, Japan) according to the manufacturers instruction.

### Statistical analysis

We used the Student’s *t*-test for comparison of the two groups and the Dunnett test for multiple comparisons. The Pearson product–moment correlation coefficient was used to evaluate association between anaphylactic response and changes in serum D-BHB concentration. Differences between measured variables were considered significant if the resultant *P* value was 0.05 or less. Analysis was performed using the JMP version 8 (SAS Institute Inc., Cary, North Carolina, USA).

## Results

### Fasting treatment attenuates the nasal hypersensitivity reaction in TDI-sensitized nasal allergy model rat

**Figure 1:**
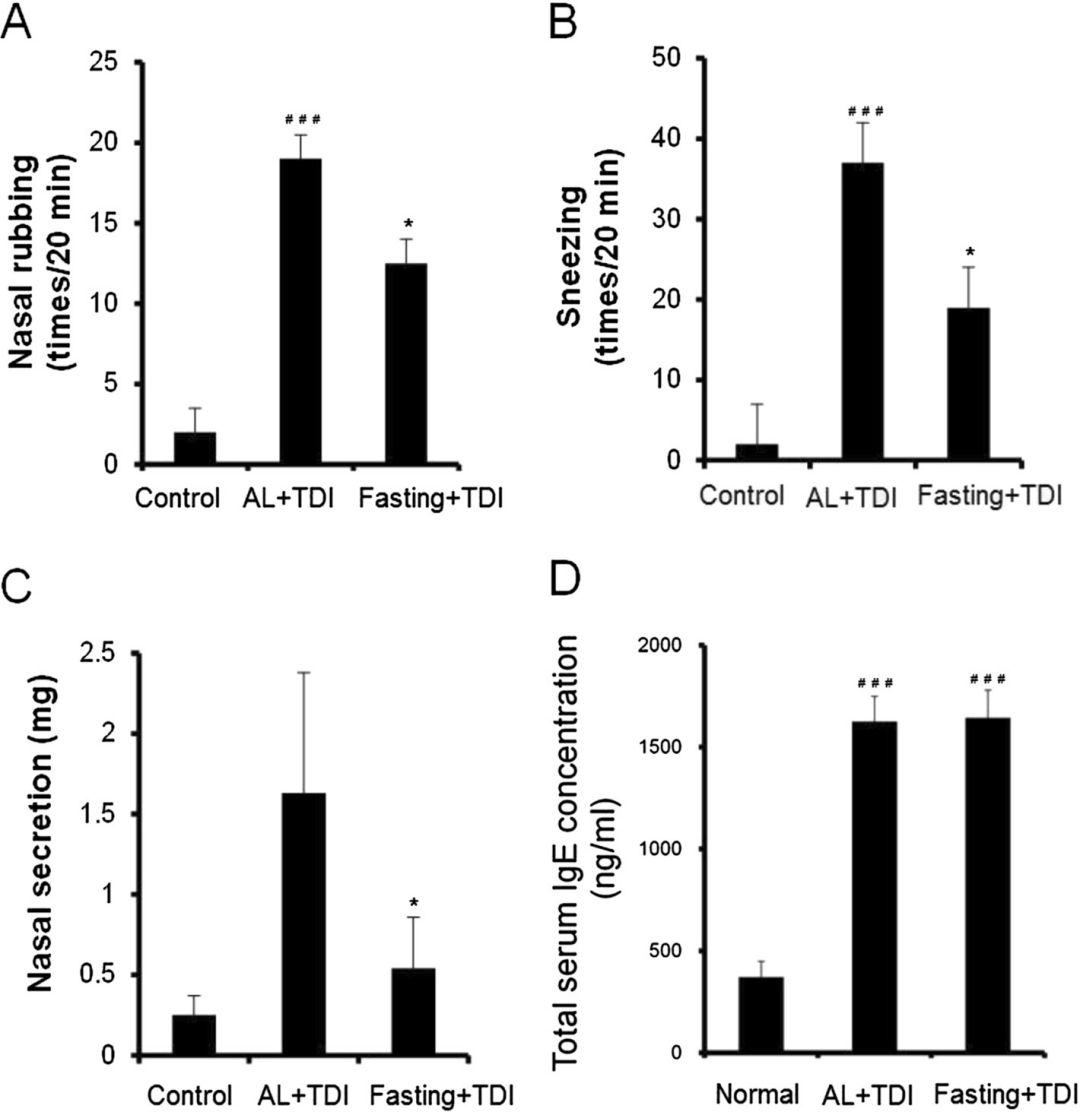
**Fasting treatment attenuates the hypersensitivity reaction in TDI-sensitized nasal allergy model rat.** Nasal rubbing **(A)**, nasal sneezing **(B)**, nasal secretion **(C)** and serum IgE concentration **(D)**. Nasal rubbing and sneezing were measured for 20 minutes immediately after provocation. Sensitized rats without applied TDI solution served as the control group. Non-sensitized rats served as the normal group. Values are means ± SEM, n = 5-6 rats, *P < 0.05 significantly different from AL + TDI. ^#^*P* < 0.05, ^##^*P* < 0.001 significantly different from control or normal group.

Figure 1 shows changes in allergy-like behaviors, nasal rubbing (A), sneezing (B), and nasal secretion (C) and serum IgE concentration (D) induced by 24 hours fasting. For the food and water *Ad libitium* group (AL), TDI application induced significant increases in each allergy behavior compared to control group. For the 24 hours fasting group, TDI application induced increases in each allergy behavior. Though, the levels were significantly lower than that of AL + TDI group.

**Figure 2:**
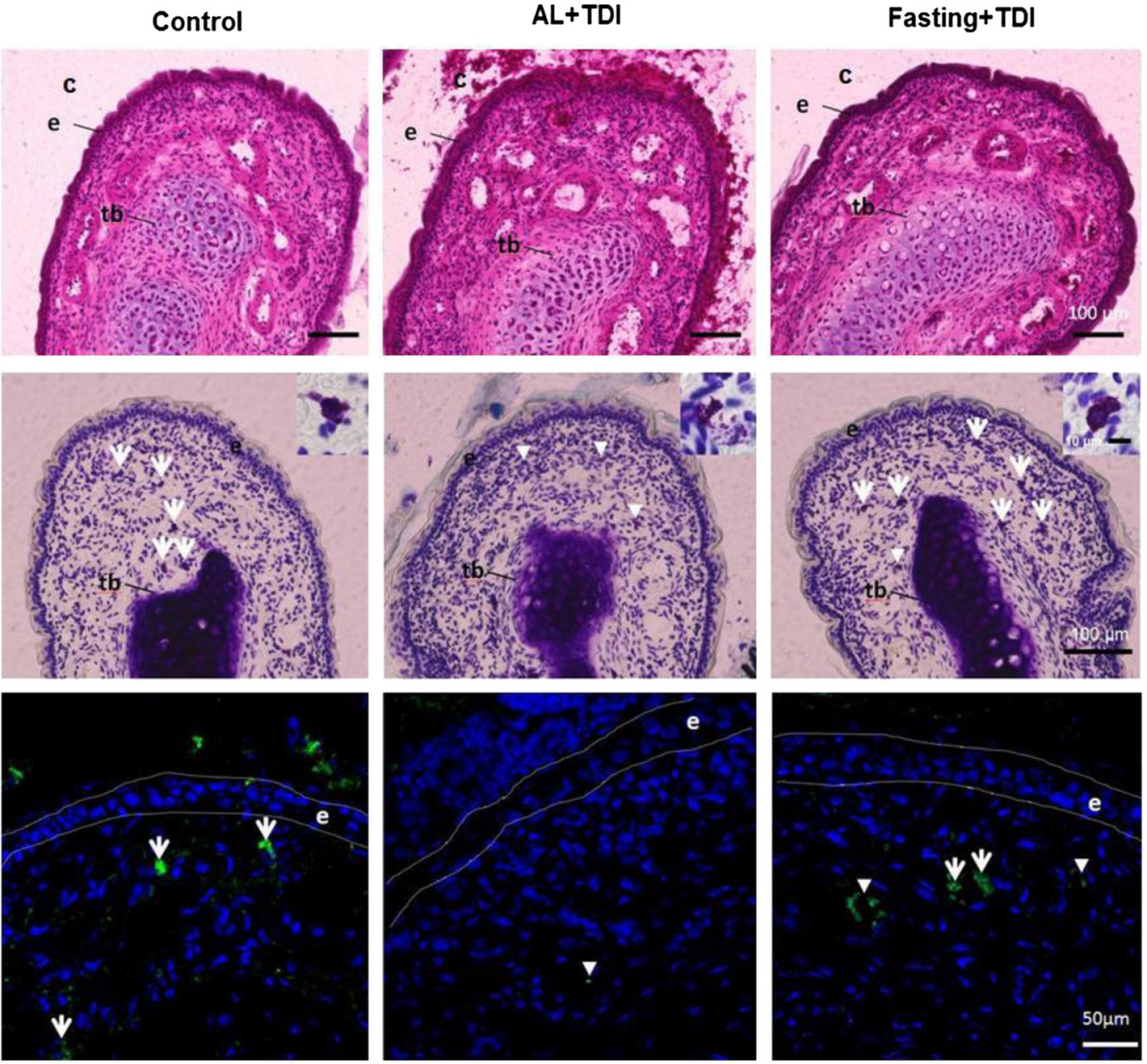
**Representative photomicrographs of the distal end of the maxilloturbinate in TDI-sensitized nasal allergy model rat.** Hematoxylin and eosin stainig (Upper). The mucus secretion in the nasal cavity and epithelia was increased in the AL with TDI. Toluidine blue staining (Middle). The degranulated mast cells presented in lamina propria of nasal epithelia in the AL with TDI. Insets show the magnified images of mast cell. Immunohistochemical detection of mast cell tryptase (Lower). Tb: turbinate bone. C: nasal cavity E: nasal epithelium. Arrow: non-degranulated mast cell. Arrow head: degranulated mast cell.

Corresponding to previous measurements of sensitization with TDI [28,29], total serum IgE concentration was significantly higher in AL with TDI group than in the normal value. For the fasting with TDI group, significant increases were observed than the normal value. Though, no significant difference was observed between AL with TDI group and fasting with TDI group.Figure 2 shows mucus secretion and mast cell alteration in the nasal cavity and mucosa of maxilloturbinate. The mucus secretion in the nasal cavity and epithelia was increased in the AL with TDI group compared to control group. In the 24 hours fasting group with TDI, these mucus secretion were maintained as same level as control group (Figure 2 upper).The mast cells presented in lamina propria of nasal epithelia were non-degranulated in the control group. In the AL with TDI group, mast cells were degranulated. Twenty four hours fasting with TDI maintained the mast cells non-degranulated (Figure 2 middle).The content of tryptase in the mast cells was apparent in the control group than AL with TDI group. In the 24 hours fasting group with TDI, mast cell tryptase were maintained as same level as control group (Figure 2 lower). These histological observations support the results of suppressed nasal hypersensitivity reaction by the fasting.

### Fasting treatment attenuates systemic anaphylaxis

**Figure 3:**
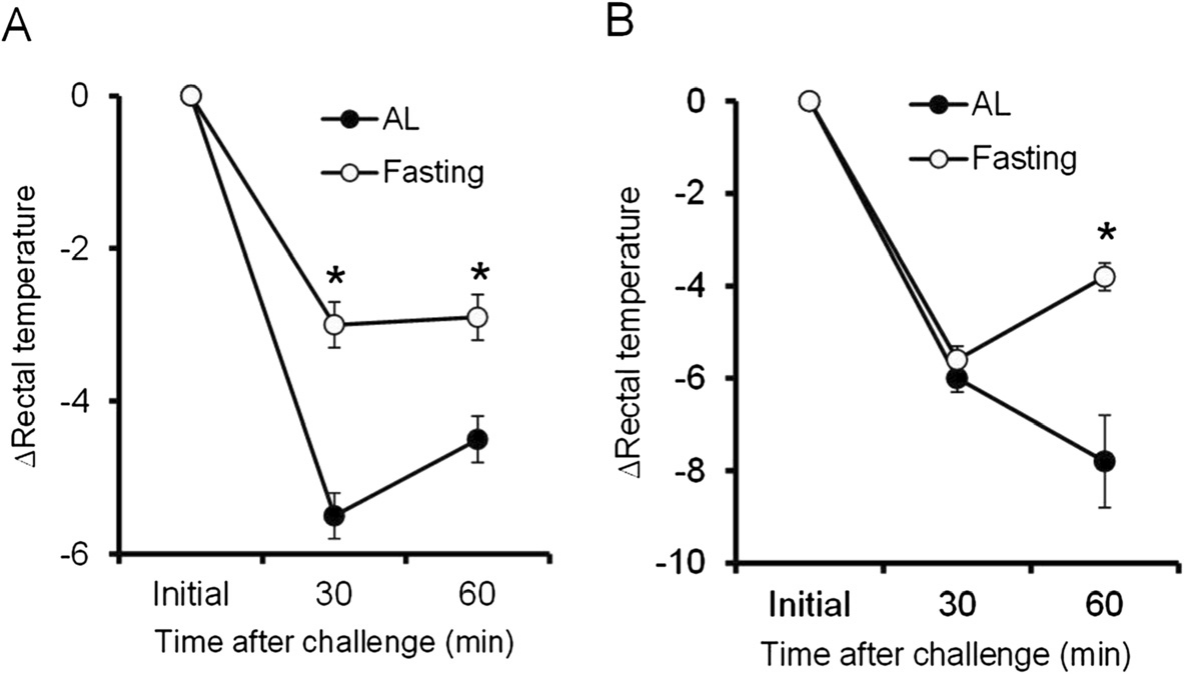
**Fasting treatment attenuates the hypersensitivity reaction in systemic anaphylaxis models.** Antigen induced IgE-dependent model **(A)**. Ig-E independent model by interperitoneal injection of compound 48/80 **(B)**. Rectal temperature was recorded after antigen or compound 48/80 provocation. Values are means ± SEM, n = 6-12 mice. *P < 0.05 significantly different from AL.

To examine the effect of food restriction on systemic anaphylaxis, we evaluated antigen or compound 48/80 induced anaphylactic responses in immediate type hypersensitivity by reductions in rectal temperature. For the 24 hours fasting group, reductions in rectal temperature were significantly smaller than those of the AL group in both antigen and compound 48/80 induced anaphylaxis models (Figures 3A and B).

### Suppression of the PCA reaction correlates with fasting duration and serum D-BHB concentrations

**Figure 4:**
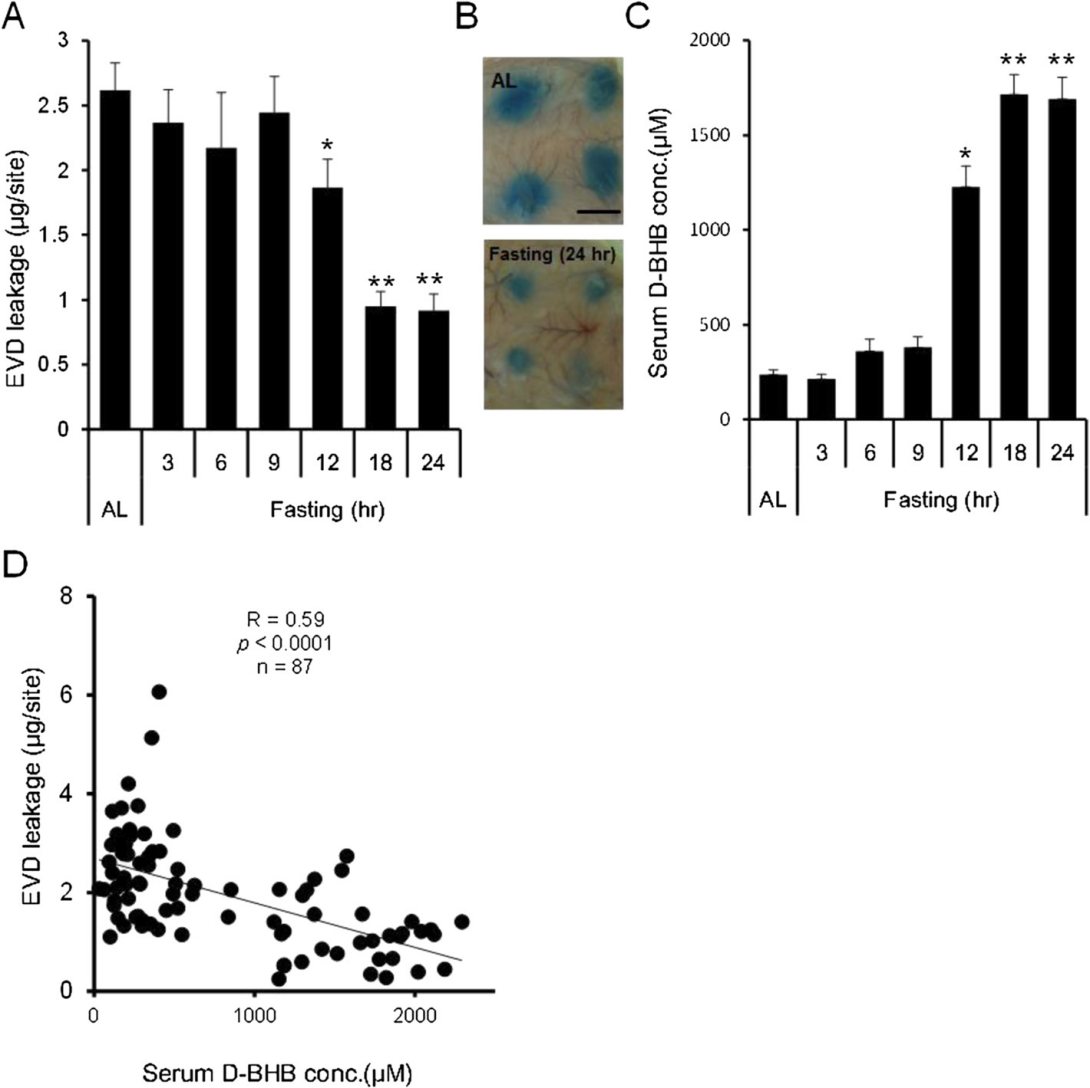
**Suppression of the PCA reaction correlate with fasting duration and serum D-BHB concentrations.** Evans blue dye (EVD) leakage **(A)**, Typical photographs of the PCA reaction **(B)**, Bar: 10 mm Serum D-BHB concentration **(C)**. Values are means ± SEM, n = 7-27 rats. *P < 0.05 significantly different from *ad libitium* (AL). Correlation between EVD leakage and D-BHB concentrations **(D)**. n = 87 rats. Fasting duration was determined as the time between food removal and antigen challenge.

It has been known that fasting markedly increases circulating concentrations of D-BHB [11]. To investigate the relationship between anaphylactic response and changes in serum D-BHB concentrations by food restriction, we quantitatively analyzed the anaphylactic response using the PCA reaction. In rats during fasting, PCA was reduced in a time-dependent manner and significant reductions were observed after 12 hours starvation (Figures 4A and B). In contrast to the PCA reaction, serum D-BHB concentrations increased in a time dependent manner and significant increases were observed after 12 hours fasting (Figure 4C). This result almost corresponds to a previous report [30]. A significant correlation between individual PCA and D-BHB concentrations was observed during the fasting period (Figure 4D).

### Relationship between changes in systemic D-BHB concentrations and inhibition of mast cell degranulation

**Figure 5:**
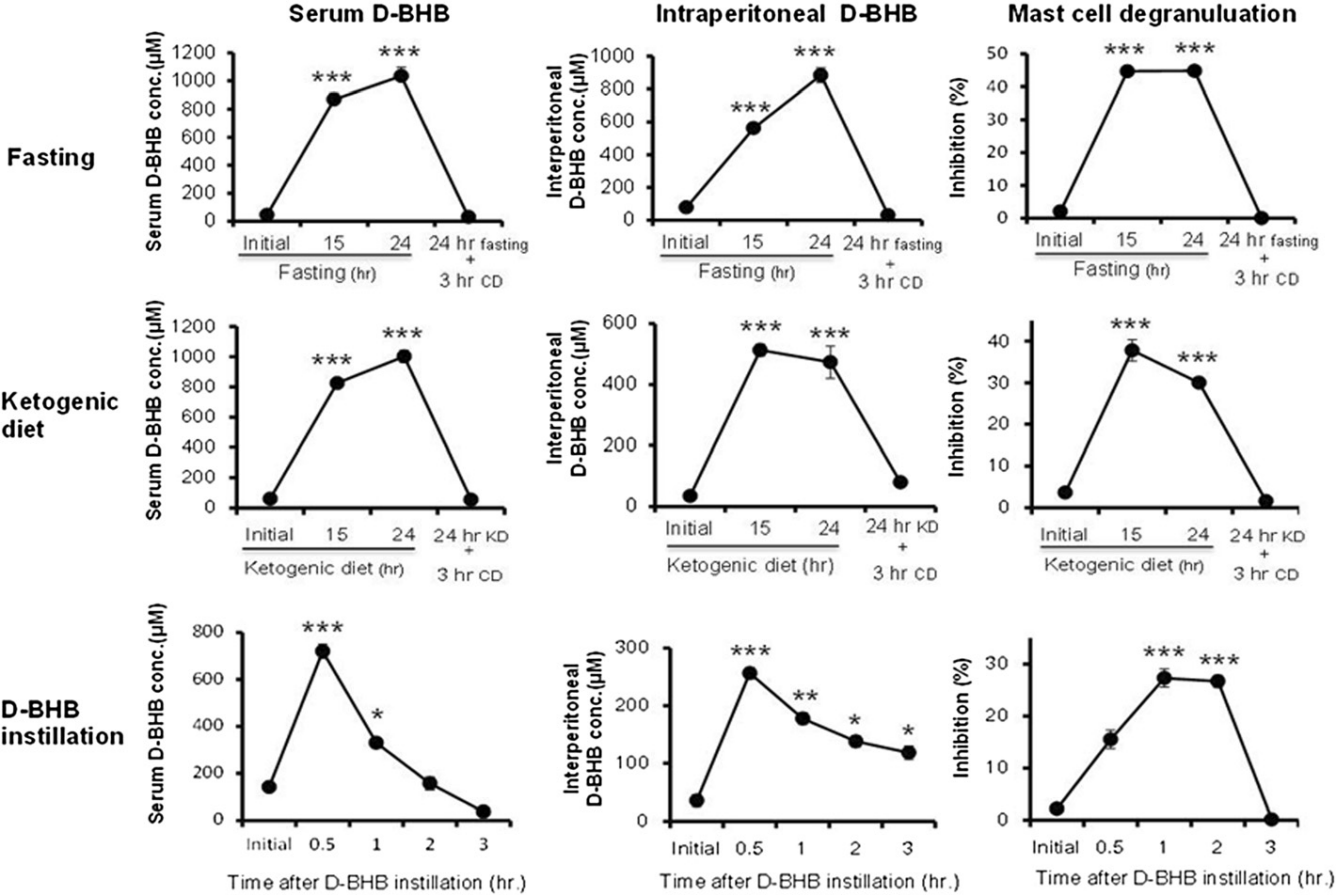
**Correlation between changes in systemic D-BHB concentrations and inhibition of mast cell degranulation.** Serum D-BHB concentration (Left), Intraperitoneal D-BHB concentration (Middle) and Inhibition of mast cell degranulation (Right). Fasting (Upper), Ketogenic diet (Middle) and Subcutaneous D-BHB instillation (Lower). Values are means ± SEM, n = 4 rats. CD: control diet. KD: ketogenic diet. *P < 0.05, **P < 0.01, ***P < 0.005 significantly different from initial values.

Degranulation of mast cells plays a crucial role in the pathogenesis of anaphylaxis and allergic disorders [22]. Two methods were used to compare the effect of elevated systemic D-BHB and mast cell degranulation with fasting; a ketogenic diet, which accelerates fatty acid oxidation; and systemic instillation of D-BHB.For fasting, significantly (approximately ten-fold) higher serum and intraperitoneal D-BHB concentrations were observed 15 and 24 hours after food restriction than that before food restriction. The elevated D-BHB concentrations returned to pre food deprivation values 3 hours after refeeding with the control diet (Figure 5 upper middle).Corresponding to changes in D-BHB concentrations, mast cell degranulation to compound 48/80 was inhibited 15 and 24 hours after food restriction and returned to pre food deprivation ratios 3 hours after the refeeding period (Figure 5 upper right).For the ketogenic diet, significantly (approximately eight-fold) higher serum and intraperitoneal D-BHB concentrations were observed 15 and 24 hours after food feeding of the ketogenic diet than that before feeding of the ketogenic diet (Figure 5 middle left and middle center). These values were almost the same as values measured with the fasting treatment. Elevated D-BHB concentrations returned to pre ketogenic diet values 3 hours after refeeding with the control diet. Corresponding to fasting and changes in D-BHB concentration, mast cell degranulation to compound 48/80 were significantly inhibited after 15 and 24 hours after food restriction and retuned to before ketogenic diet ratio 3 hours after re-feeding (Figure 4 middle right).For intradermal infusion of D-BHB, significant increases in serum and intraperitoneal D-BHB concentrations were observed immediately after infusion, which then gradually decreased (Figures 5 lower left and lower center). Serum D-BHB returned to initial levels after 3 hours. Intraperitoneal D-BHB concentrations remained at approximately half the value of the maximal concentrations after 3 hours infusion. Mast cell degranulation was significantly inhibited after D-BHB infusion and the inhibition rate plateaued 1 and 2 hours after infusion. Inhibitory effect was diminished 3 hours after infusion (Figure 5 lower right).

### Fasting and D-BHB suppresses mast cell degranulation and intracellular calcium oscillations by compound 48/80

Changes in [Ca^2+^]_i_ responses to compound 48/80 were analyzed in fura 2-loaded RPMC derived from the ketogenic diet, fasting, and in vitro exposure to 100 mM D-BHB.

**Figure 6:**
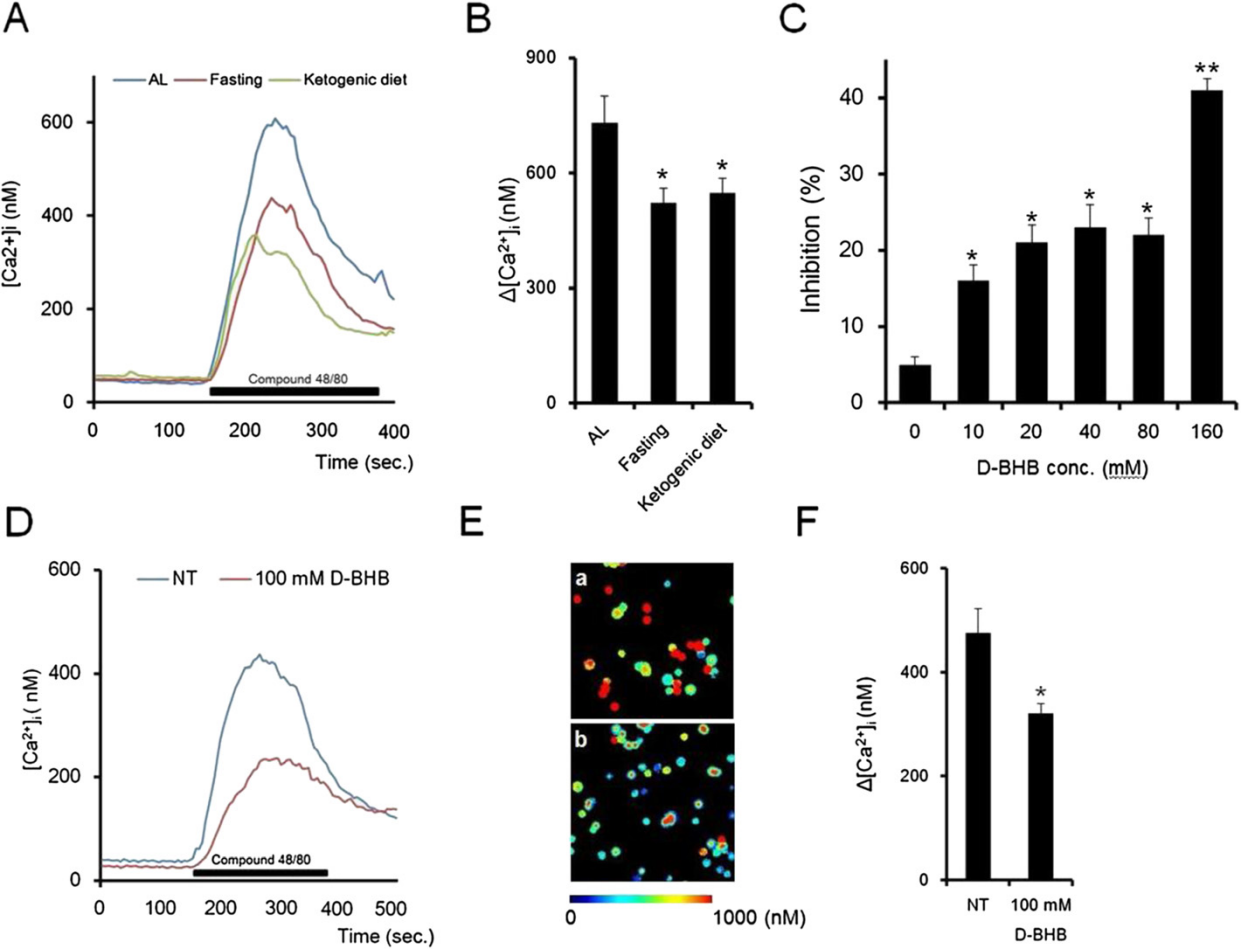
**Fasting and D-BHB suppress the RPMC [Ca**^**2+**^**]i mobilization response to compound 48/80.** Time courses for [Ca^2+^]i responses of mast cells from fasting and the ketogenic diet **(A)**. Values represent means (n = 12 aciner cells). Comparison of [Ca^2+^] i changes in response to compound 48/80 **(B)**. RPMC prepared form each treatment were stimulated with 1 μg/mL compound 48/80 for 240 sec. Values are means ± SEM, n = 12 aciner cells, *P < 0.05 significantly different from *ad libitium* (AL). The effect of D-BHB on mast cell degranulation **(C)**. Cells were preincubated with various concentrations of D-BHB for 10 minutes, and then incubated (10 minutes) with 1 μg/mL compound 48/80. Values are means ± SEM, n = 6 rats. Time courses for [Ca^2+^]i responses of mast cells treated with D-BHB **(D)**. Values represent means (n = 12 aciner cells). **E**: Pseudo color [Ca^2+^]i images of non-treatment **(a)** and D-BHB treatment **(b)** mast cells activated by compound 48/80. The effect of D-BHB on [Ca^2+^]i changes in response to compound 48/80 **(F)**. Cells were stimulated with 1 μg/mL compound 48/80 for 240 sec under 100 mM D-BHB. Values are means ± SEM, n = 6, * *P* < 0.05 significantly different from non-treatment (NT).

Compound 48/80 evoked increases in [Ca^2+^]_i_ upon a 240 seconds application and these responses were reversible. Compare to RPMC The [Ca^2+^]_i_ response to compound 48/80 was lower in RPMC derived from the ketogenic diet and fasting group than RPMC derived from free access to the control diet. The amplitude of the [Ca^2+^]_i_ increase in fasting was similar to that obtained with the ketogenic diet (Figure 6A). The peak increase in [Ca^2+^]_i_ from baseline (Δ[Ca^2+^]i) was significantly lower in the ketogenic diet and fasting group than that in the control diet group (Figure 6B).

To confirm the effect of D-BHB, we incubated D-BHB with isolated mast cell in vitro*.* D-BHB inhibited mast cell degranulation in a dose dependent manner (Figure 6C). Significant inhibition was observed at more than 10 mM (Figures 6D and E). In addition, pretreatment with D-BHB decreased compound 48/80 evoked [Ca^2+^]_i_ and Δ[Ca^2+^]_i_ was significantly suppressed (Figure 6F).

## Discussion

The present study clearly demonstrated that food deprivation attenuated local and systemic immediate hypersensitivity symptoms. This attenuation was significantly correlated with the duration of food restriction and increases in blood D-BHB concentrations (Figures 4 and 5). Type I allergy is an immediate hypersensitivity reaction that is characterized by allergen-induced mast cell degranulation and is followed by the immediate release of the chemical mediator histamine from mast cells stored in vesicles, which leads to inflammation, smooth muscle contraction, and vasodilation reaction [31]. In the present study, fasting did not affect serum IgE concentration (Figure 1C). D-BHB is an abundant ketone body normally circulating in the blood, but an antihistamine effect or antagonistic action to immune reactive chemical mediators has not been reported [31]. Therefore, to further investigate the relationship between D-BHB and allergic reactions, we focused on the effect of D-BHB on mast cell activation.

It has been reported that, the restriction of nutrition supplementation creates various enhancing effect on the cell mediated defense. To exclude the possible defense response by food deprivation other than increased D-BHB, we employed two methods to elevate internal D-BHB concentration in addition to fasting; a ketogenic high-fat, adequate-protein, and low-carbohydrate diet that accelerates fatty acid oxidation [32] and systemic instillation of D-BHB. Our results showed that, both treatments dramatically increased blood and intraperitoneal D-BHB concentrations similar to fasting and this was accompanied by inhibition of mast cell degranulation induced by compound 48/80 (Figure 5). The inhibitory effect of mast cell degranulation by each treatment was diminished after D-BHB concentrations returned to a normal state by the interruption of each diet and D-BHB infusion. From these observations, we speculated that endogenous D-BHB which was dramatically increased by food deprivation, plays an important role in the attenuation of hypersensitivity symptoms through suppression of mast cell activation.

Increases in intracellular calcium activity are critical for the rapid exocytotic response of mast cells. The signaling pathway leading to exocytosis of mast cells has been well characterized [33]. Interestingly, mast cells delivered from 24 hours of food deprivation and a ketogenic diet showed a decrease in mast cell [Ca^2+^]_i_ mobilization induced by exposure to compound 48/80. Furthermore, in vitro D-BHB treatment with mast cells delivered from a control diet rat also suppressed degranulation and [Ca^2+^]_i_ mobilization (Figure 4). G protein coupled receptor-mediated activation of phospholipase C and the associated production of inositol 1,4,5-trisphosphate induces release of Ca^2+^ from stores in the endoplasmic reticulum and Golgi apparatus through Ca^2+^-conducting IP_3_-receptors. Compound 48/80 is also known to initiate mast cell degranulation through direct activation of G proteins, which was proposed to mimic receptor signaling [34,35]. Thus, we speculate that D-BHB might have inhibited downstream of G-protein coupled intracellular signaling pathway, which leads to stabilization of mast cell membranes against degranulation stimuli. Further study is necessary to determine the cellular and molecular mechanism(s) underlying the stabilization of mast cells by D-BHB.

During a fasting state, D-BHB is synthesized in the liver from acetyl-CoA mostly derived from beta-oxidation of fatty acids. The rate limiting step of D-BHB formation is mitochondrial hydroxymethylglutaryl-CoA synthase (mHMG-CoAS), which converts acetoacetyl-CoA and acetyl-CoA into 3-hydroxy-3-methylglutaryl-CoA [36]. The enzymatic activity of HMG-CoAS is elevated by deacetylation by sirtuin-3 (Sitrt3) [37]. Also, the mammalian target of the rapamycin (mTOR) signal controls D-BHB formation during fasting by modulating peroxisome proliferator-activated receptor mediated activation of the mHMG-CoAS enzyme [38]. Sirt3 and mTOR are known as key regulators of energy metabolic homeostasis during fasting and calorie restriction [39,40], which has been linked to ameliorate diseases associate with aging [41,42]. Several studies have shown that calorie restriction can mitigate excessive immune response [43-46], but the mechanisms for this attenuation are still largely unknown. Prolonged restriction of calorie intake also leads to higher serum D-BHB concentrations [47-49]. Altogether, our findings with the pleiotropic property of D-BHB were able to rule out the possibility that D-BHB is not the only alternative energy source that spares glucose, but is one of the defense molecules that participates in a number of health benefits during dietary restriction.

## Conclusion

We demonstrated that fasting attenuated immediate hypersensitivity symptoms. In addition, we provided evidence that D-BHB generated by fasting plays an important role for the sedate excess immune response against immediate hypersensitivity. We believe further investigations focused on the role of ketone bodies in immunological function will pave the way to understanding the molecular mechanisms for the positive health benefits of fasting.

## Competing interests

The authors declare that they have no competing interests.

## Authors’ contributions

SN, IS and KT conceived the project. KT, IS, RH and SN supervised all research. SN, RH, IS and KT wrote the manuscript. SN and RH prepared figure. SN, RH, SS and IS designed the experiments. SN, RH and SS performed the experiments. All authors read and approved the final manuscript.
